# Altered Brain Functional Connectivity and Peripheral Transcriptomic Profiles in Major Depressive Disorder With Childhood Maltreatment

**DOI:** 10.1155/da/6059502

**Published:** 2025-03-31

**Authors:** Min Wang, Jinxue Wei, Yushun Yan, Yue Du, Huanhuan Fan, Yikai Dou, Liansheng Zhao, Rongjun Ni, Xiao Yang, Xiaohong Ma

**Affiliations:** Mental Health Center and Institute of Psychiatry, West China Hospital of Sichuan University, Chengdu, Sichuan, China

**Keywords:** childhood maltreatment, functional connectivity, major depressive disorder, transcriptome

## Abstract

**Background:** Childhood maltreatment (CM) is a significant risk factor for major depressive disorder (MDD), yet the underlying biological mechanisms remain unclear. This study aimed to investigate brain functional networks and peripheral transcriptomics in patients with MDD who have a history of CM.

**Methods:** Functional imaging data were collected and network-based statistics were used to identify differences in functional networks among MDD patients with CM (MDD_CM, *n* = 78), MDD patients without CM (MDD_nCM, *n* = 61), and healthy controls (HC, *n* = 126). Additionally, blood transcriptional data were clustered into co-expression modules, and module differential connectivity analysis was utilized to assess variations in gene co-expression network modules among the groups.

**Results:** The results revealed a significant difference in an inferior occipital gyrus-centered functional network among the three groups. Furthermore, eight gene co-expression modules differed among the groups and were enriched in multiple branches related to immune responses or metabolic processes. Notably, a module enriched in type I interferon-related signaling pathways demonstrated a significant correlation with the disrupted network in the MDD_nCM group. Moreover, multiple immune-related gene modules were found to be significantly correlated with sleep disturbances in MDD_CM patients.

**Conclusions:** Dysregulation of an inferior occipital gyrus-centered functional network and immune-related transcriptomic alterations significantly associate with the pathophysiology of MDD_CM.

## 1. Introduction

Major depressive disorder (MDD) is a complex and heterogeneous mental disorder that affects more than 280 million people annually worldwide, resulting in substantial economic and healthcare burdens across communities [1]. Childhood maltreatment (CM), including emotional, physical, or sexual abuse, as well as neglect, has consistently been reported to be linked with the development and persistence of MDD [2]. Individuals who have experienced CM often face long-lasting impairments in multiple biological functions, such as systemic inflammation, immune dysfunction, and alterations in brain structure and function [3]. Previous research has suggested that MDD with a history of CM (MDD_CM) should be recognized as a specific clinical phenotype of MDD with a more severe disease trajectory [4]. However, the biological mechanisms underlying MDD_CM have not yet to be fully elucidated.

Numerous neuroimaging studies have suggested that CM is associated with changes in the functional brain network, particularly those affecting regions involved in cognition, emotion, and reward processes [5]. Patients with MDD_CM also was demonstrated changes in resting-state functional connectivity (FC), including the amygdala [6], hippocampal [7], thalamic [8] and prefrontal-limbic-cerebellar circuit [9, 10] compared with MDD patients without CM (MDD_nCM). However, these studies focused primarily on specific brain regions when FC was examined, ignoring the fact that the brain is a highly interconnected network referred to as the “human brain connectome” [11]. Thus, focusing on the whole brain network would be helpful to gain more insight into the brain functional network in MDD_CM [12].

The transcriptome provides a genome-wide assay of biological mechanisms by measuring RNA expression levels. Previous studies have demonstrated that gene transcriptomic regulation is related to CM and MDD pathogenesis [13–15]. To the best of our knowledge, only two studies conducted on the same cohort have investigated the associations between CM and alterations in RNA expression in patients with MDD. One study examined the global impact of CM through factorial analysis but did not obtain any significant results in relation to CM and RNA sequencing in patients with MDD [14]. Another study using principal component and gene-set enrichment analyses revealed that emotional abuse was associated with proinflammatory responses through the dysregulation of RNA transcriptome expression, particularly in the immune system [16]. These inconsistent results warrant further efforts to explore the transcriptomic profiles of MDD_CM.

Network-based methodologies have significantly improved our understanding of transcriptional profiles in MDD. Network-based statistics (NBS) [12] and weighted gene co-expression network analysis (WGCNA) [17] are both network-based methods that are widely applied in the analysis of brain networks and transcriptional profiles, respectively. By evaluating the overall edges of the brain as a unified system, the NBS significantly enhances statistical power compared with the methods that address multiple comparisons across individual edges [12]. Moreover, WGCNA clusters genes with similar functions into a module, revealing the complex relationships among multiple genes related to neuropsychiatric disorders [17]. Employment of multidimensional strategies, including combinations of transcriptomic and brain images, has been applied in the studies of CM [18] and MDD [19], which significantly promotes our understanding of the mechanism of CM and MDD. Therefore, combined analysis of FC and transcriptional profiles would be helpful to better understand the pathophysiology of MDD_CM.

In this study, we characterized network-based features of the FC brain network and transcriptional profiles, and combined these features to explore the underlying biological mechanisms of the pathophysiology of MDD_CM.

## 2. Materials and Methods

### 2.1. Participants

**Figure 1:**
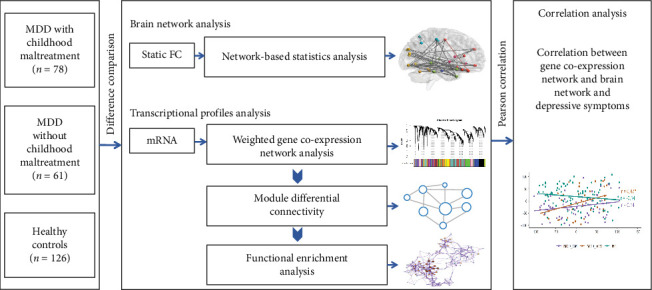
Flow diagram. FC, functional connectivity; MDD, major depressive disorder.

A total of 265 participants were recruited from the Mental Health Center of West China Hospital of Sichuan University from January 2016 to December 2020. Three groups of participants, aged 16–60 years, were established (see Figure 1 and Figure S1): (1) patients with MDD with a history of CM (MDD_CM, *n* = 78), (2) patients with MDD without a history of CM (MDD_nCM, *n* = 61), and (3) individuals with no history of any Diagnostic and Statistical Manual of Mental Disorders IV (DSM-IV) Axis I disorders, CM or usage of psychotropic medication (HC, *n* = 126). The inclusion and exclusion criteria were in line with those of previous publications [19, 20]. All patients met the DSM-IV criteria for MDD and had no comorbid Axis I or II disorders. Patients who had used any antidepressants or antipsychotics within 3 months preceding recruitment were excluded. Additionally, both patients and HC with neurodegenerative diseases, major endocrine or metabolic disorders, or those who were pregnant or breastfeeding were also excluded from the study. Participants spent approximately 2 h completing all the assessments. The research protocol received ethics approval from the Institutional Ethics Committee of West China Hospital, Sichuan University (Approval Number: [2016] 170). Each participant was fully informed about the study and provided written consent.

The Hamilton Anxiety Scale (HAMA) [21] was used to assess anxiety symptoms, whereas the Hamilton Depression Scale (HAMD) [22] was administered to evaluate depressive symptoms in all the patients with MDD. The HAMA has two subscales: mental anxiety (items 1–5) and somatic anxiety (items 6–14). The HAMD includes five subscales: retardation (items 1, 7, 8, 14), cognitive disturbance (items 2, 3, 9), sleep disturbance (items 4, 5, 6), somatization (items 10, 11, 12, 15, 17), and weight (item 16).

### 2.2. Assessment of Childhood Maltreatment

CM was assessed using the Childhood Trauma Questionnaire (CTQ), a widely validated self-report instrument designed to measure the early-life trauma in individuals based on their experiences before the age of 16 [23]. The CTQ consists of five subscales, each evaluating a specific form of CM: emotional abuse (EA), physical abuse (PA), sexual abuse (SA), emotional neglect (EN), and physical neglect (PN). To classify participants into MDD_CM and MDD_nCM groups, we adopted predefined cutoff scores for each CTQ subscale, as established in prior research [23]. Specifically, the cutoff values were set at EA ≥13, PA ≥10, SA ≥8, EN ≥15, and PN ≥10. In this study, participants were categorized as having experienced CM if their score met or exceeded the threshold in at least one of these subscales [7, 24].

### 2.3. MRI Data Acquisition and Analysis of the Functional Network

All participants in this study underwent brain imaging via a 3-Tesla whole-body MR scanner (Achieva, Philips, Netherlands) that equipped with an eight-channel phased-array head coil. For functional imaging, an echo-planar imaging (EPI) sequence was used, with the following parameters: time repetition (TR) = 2000 ms, echo time (TE) = 30 ms, flip angle = 90°, 38 slices, in-plane matrix size = 64 × 64, field of view (FOV) = 240 mm^2^ × 240 mm^2^, and voxel dimensions = 3.75 mm^3^ × 3.75 mm^3^ × 4 mm^3^. In addition, for T1 imaging, the parameters were time repetition (TR) = 8.37 ms, echo time (TE) = 3.88 ms, field of view (FOV) = 24 cm^2^ × 24 cm^2^, flip angle = 7°, in-plane matrix resolution = 256 × 256, voxel size = 1 mm isotropic, and 188 slices.

Functional images were preprocessed using Statistical Parametric Mapping software (SPM12, v 6225) and the Data Processing Assistant for Resting-State fMRI (DPARSF, v 5.3) both in MATLAB 2018a (MathWorks, Natick, MA, USA). The first 10 scans were excluded, followed by slice-timing and head motion correction for the remaining functional scans. The EPI images were then spatially normalized to the Montreal Neurological Institute (MNI) template, with each voxel resampled to 3 mm^3^ × 3 mm^3^ × 3 mm^3^. To induce low-frequency drift and high-frequency physiological noise, a temporally bandpass filter (0.01–0.08 Hz) was applied. Head motion was addressed using the Friston 24-parameter model, and signals from white matter (WM) and cerebrospinal fluid (CSF) were excluded to focus the analysis on gray matter voxels. Notably, global signal regression (GSReg) was not performed to avoid potential biases in regional correlations resulting from the interregional correlation patterns. In the present study, all participants exhibited <2 mm displacement and 2° of rotation in any direction, and the mean framewise displacement (FD) was <0.2 mm at each time point.

Network nodes were defined on the basis of each region of interest (ROI) from the automated anatomical labeling (AAL3) atlas [25]. The mean time series data of these ROIs were used to calculate Pearson's correlations between different regions. This process generated a set of 164 × 164 correlation connectivity matrices for each subject, summarizing the patterns of interregional connectivity within the brain.

The NBS was used to analyze the significant statistical FC network between different groups using the R package NBR [12]. An ANOVA model was used to compare the network among the MDD_CM, MDD_nCM, and HC groups. The parameters were set as follows: primary *p* threshold = 0.001, random permutation = 5000. The results were visualized using the BrainNet Viewer (v 1.61) [26] and R package circlize (v 0.4.15) [27]. Linear models with head motion, age, sex, and education years as covariates were used to compare the average FC between the three sample groups. Kolmogorov‒Smirnov tests were applied to estimate the differences in the distributions of Fisher's *r*-to-*Z* transformed FC.

### 2.4. Peripheral Blood Collection and RNA Sequencing

Upon enrollment, peripheral blood samples were collected from the participants (51 MDD_CM, 37 MDD_nCM, and 89 HC). These samples were collected via Tempus Blood RNA Tubes (Applied Biosystems, Foster City, CA), and total RNA was extracted via the MagMAX for Stabilized Blood Tubes RNA Isolation Kit (Thermo Fisher Scientific, Waltham, MA, USA) following the manufacturer's guidelines. The isolated RNA was preserved at −80°C until further use.

### 2.5. RNA Sequencing and Data Processing

For RNA sequencing, 1 μg of RNA served as the starting material. Messenger RNA (mRNA) was isolated from the total RNA extracts using oligo (dT) beads, followed by fragmentation of the mRNA into 200–500 nucleotide (nt) segments using a specific buffer. The fragmented RNA was then reverse transcribed to synthesize the first strand complementary DNA (cDNA), which was subsequently complemented with the second strand cDNA via DNA polymerase I. Following purification, the double-stranded cDNA was subjected to end repair, dA addition, and ligation with Illumina sequencing adapters. PCR amplification was subsequently carried out to generate the cDNA library. The library was sequenced on a NovaSeq 6000 System (by Illumina) using a 2 × 150 base pair (PE) setup according to the manufacturer's guidelines. Raw reads obtained from sequencing were processed through Cutadapt (v1.9.1) for filtration, generating clean reads. These cleaned reads were then aligned to the reference genome using HISAT2 software (v 2.0.1).

To filter out lowly expressed transcripts, those with counts per million (CPM) less than one in more than 5% of total samples were discarded. The trimmed mean of M-values (TMM) normalization method was subsequently applied. To assess the sample outliers and overall relationships, a biological coefficient of variation (BCV) plot was generated. Dispersions were estimated using the quantile-adjusted conditional maximum likelihood (qCML) approach.

### 2.6. Constructed Gene Co-Expression Network and Module Preservation Analysis

The co-expression network of genes and module preservation analysis were constructed using the R package WGCNA [28]. The MDD_CM sample was used as the reference dataset. The RNAs whose expression was highly correlated in the reference dataset were clustered as co-expression modules and named arbitrarily. Modules represent clusters of highly interconnected genes, characterized by strong absolute correlations between them. The soft thresholding power was chosen based on the criterion of approximate scale-free topology. The construction of co-expression modules involved setting a minimum module size of 30 and a clustering height cutoff of 0.1.

Module preservation statistics were used to evaluate whether gene co-expression modules were preserved in MDD_CM, MDD_nCM, and HC groups. Module preservation statistics were developed to determine which aspects of within-module topology are preserved in different datasets. A composite preservation statistic (*Z*_summary_) was used to evaluate of module preservation in this study. *Z*_summary_ is the average of the density-based preservation statistics and the connectivity-based preservation. A *Z*_summary_ score higher than 10 indicates strong evidence of preservation, a *Z*_summary_ score between 2 and 10 indicates weak to moderate evidence of preservation, and a *Z*_summary_ score less than 2 indicates no evidence of preservation [29].

### 2.7. Module Differential Connectivity

The module differential connectivity (MDC) method, implemented in the R package DGCA, was used to examine differences in gene co-expression modules between groups (MDD_CM vs., MDD_nCM, MDD_CM vs., HC, MDD_nCM vs., HC). This method focuses on contrasting the connectivity ratio between all gene pairs within a module across groups, which quantifies the intramodular connectivity strength. An MDC value above 1 indicates an increase in connectivity (GOC) or heightened gene co-expression, whereas values below 1 indicate a decrease in connectivity (LOC) or diminished co-expression among genes [30, 31].

### 2.8. Functional Enrichment Analysis

To explore the biological functions of the genes in the identified modules, the Gene Ontology (GO) and Kyoto Encyclopedia of Genes and Genomes (KEGG) enrichment analyses were performed using the *R* package clusterProfiler (v 4.10). Terms with FDR-corrected *p* values of <0.05 were considered significantly enriched within modules.

### 2.9. Protein–Protein Interaction Network and Hub Genes

To identify potential relationships among the proteins encoded by the genes in the module, we used the Search Tool for the Retrieval of Interacting Genes (STRING) database (v 12.0) to construct a protein−protein interaction (PPI) network and used Cytoscape (v 3.9.1) to visualize the network. Additionally, the 10 hub genes with the highest degree of connectivity in the PPI network were identified using the CytoHubba plugin.

### 2.10. Statistical Analysis

Statistical analysis was performed using the R software environment. The chi-square test was applied to compare sex differences, and one-way ANOVA was used to compare age and years of education among MDD_CM, MDD_nCM, and HC groups. The difference in total disease duration between MDD_CM and MDD_nCM groups was tested using Student's *t*-test. The Wilcoxon test was used to compare differences in HAMA scores and HAMD scores between MDD_CM and MDD_nCM groups. Spearman's correlation analysis was applied to explore the relationships between FC, gene co-expression network modules, and depressive symptoms, while controlling for age, sex, and education as covariates. Fisher's *Z* transformation was applied to compare the correlation coefficients between the MDD_CM and MDD_nCM groups by converting them into *Z* values and performing hypothesis testing under an approximate normal distribution.

## 3. Results

### 3.1. Demographic Characteristics

A total of 78 MDD_nCM patients, 61 MDD_CM patients, and 126 HC were included in this study. There were no significant differences in age, sex, or education years among the three groups. In addition, there were no significant differences in the number of episodes, total duration of disease, or scores on the HAMD and HAMA scales between MDD_CM and MDD_nCM groups (Table 1). All participants were included in the functional connectivity analysis, whereas 177 individuals (MDD_CM, *n* = 51; MDD_nCM, *n* = 37; HC, *n* = 89) were included in the transcriptional profile analysis (see Table S1).

### 3.2. Differences in the Brain Functional Network Among the MDD_CM, MDD_nCM, and HC Groups

**Figure 2:**
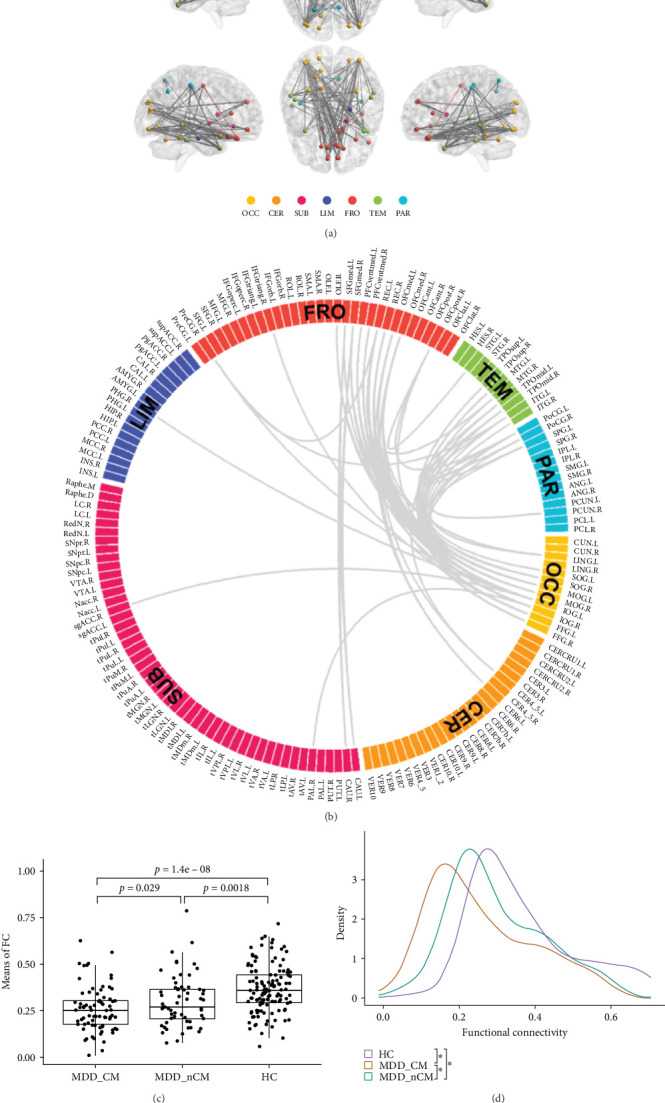
Functional network differences between the MDD_CM, MDD_nCM, and HC groups. (A) A functional network containing 40 nodes and 83 edges was different among MDD_CM, MDD_nCM, and HC groups. (B) The functional network differences among MDD_CM, MDD_nCM, and HC groups were presented in circular visualization. (C) Differences in network-averaged functional connectivity between MDD_CM, MDD_nCM, and HC groups. (D) Distribution of edge-weights (i.e., group-averaged functional connectivity) of the functional network in MDD_CM, MDD_nCM, and HC groups. CER, cerebellum; FRO, frontal lobe; HC: healthy control; LIM, limbic system; MDD_CM, major depressive disorder with childhood maltreatment; MDD_nCM, major depressive disorder without childhood maltreatment; OCC, occipital lobe; PAR, parietal lobe; SUB, subcortical structures; TEM, temporal lobe.

The results of the NBS analysis revealed that an IOG-centered network comprising 40 nodes and 83 edges, exhibited significant differences across the three groups (see Table S2). The majority of nodes in the network were identified in the visual network, default mode network (DMN), central executive network (CEN), limbic system, and cerebellum. Notably, the bilateral inferior occipital gyrus (IOG) emerged as the most connected node with 27 edges, followed by the middle occipital gyrus (MOG) with 19 edges, the rectus gyrus (REC) with 14 edges, and the medial orbital gyrus (OFCmed) with 11 edges. In addition, the medial superior frontal gyrus (SFGmed), medial orbital part of the superior frontal gyrus (PFCventmed), middle temporal gyrus (MTG), inferior temporal gyrus (ITG), and temporal pole (TPOmid) were also highly connected nodes. Other nodes in the network included the caudate nucleus, right subgenual anterior cingulate cortex (sgACC), the left lobule IV, V (CER4_5), and VI (CER_6) of the cerebellar hemisphere (Figure 2A, B). The average value of all edges in this network was found to be lower in the MDD_CM group than in the MDD_nCM (*p* = 0.028) or HC (*P* = 1.42 × 10^−8^) groups. Additionally, the average value was also lower in the MDD_nCM group than in the HC group (*p* = 0.002). The distribution of functional connectivity across all edges of this network was observed to be more negative in the MDD_CM group than in the MDD_nCM group (*D* = 0.313, *p* = 0.0005) or HC group (*D* = 0.458, *p* = 3.22 × 10^−8^). Similarly, it was also more negative in the MDD_nCM group than in the HC group (*D* = 0.313, *p* = 0.0005) (Figure 2C, D).

Furthermore, general linear regression analysis in the MDD group revealed significant negative correlations between the brain network and CM (total scores of CTQ: *β* = −0.002, *p* = 0.001; EA: *β* = −0.005, *p* = 0.018; EN: *β* = −0.006, *p* < 0.001; PN: *β* = −0.008, *p* = 0.002; SA: *β* = −0.015, *p* = 0.043) see Table S3).

### 3.3. MDC Highlights Gene Co-Expression Modules that Differed Among Groups

**Figure 3:**
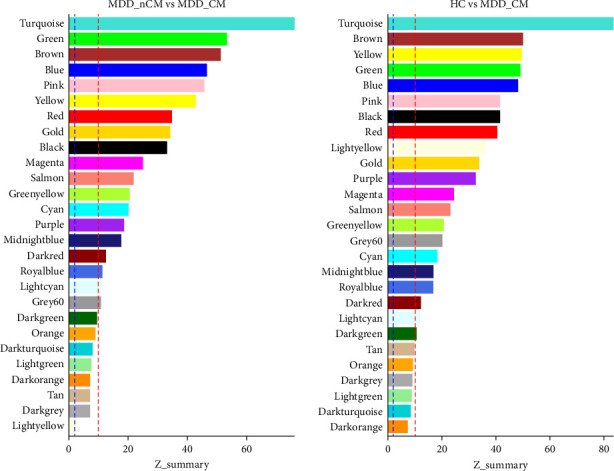
Preservation of co-expression modules between the sample groups. The preservation of co-expression modules between different sample groups was evaluated using a summary preservation statistic (*Z*_summary_). The blue dashed line indicates *Z*_summary_ of 2, and the red dashed line indicates *Z*_summary_ of 10. HC, healthy control; MDD_CM, major depressive disorder with childhood maltreatment; MDD_nCM, major depressive disorder without childhood maltreatment.

After alignment with the reference genome, a total of 61806 mRNA transcripts were successfully identified. Following the filtering process to remove those with low expression, 11708 mRNAs were selected for further analysis. The co-expression modules were then clustered across the three groups, and the soft thresholding power was set to 7 based on the criterion of approximate scale-free topology. In total, 27 gene modules comprising 38–2260 genes were clustered. Each module was assigned a unique color for easy identification. A grey module was created to group 296 mRNA transcripts that could not be clustered into any of the co-expression modules (see Figure S2, Table S4). We found that transcriptional structures in the modules were preserved (*Z* summary score >2) in both the MDD_nCM and HC groups (Figure 3, see Table S5).

The differences in connectivity of modules among groups were subsequently analyzed (Table 2), revealing significant differences were observed between MDD_CM and MDD_nCM in the red module (MDC = −0.726, *p* = 0.022), grey60 module (MDC = −0.7, *p* = 0.035), and midnightblue module (MDC = 1.203, *p* = 0.019). Additionally, the grey60 module (MDC = 1.383, *p* = 0.043) and midnightblue module (MDC = −0.794, *p* = 0.014) differed between MDD_nCM and HC. Furthermore, there were significant differences between MDD_CM and HC in the darkred module (MDC = 0.792, *p* = 0.019), darkturquoise module (MDC = 0.645, *p* = 0.035), lightcyan module (MDC = 0.713, *p* = 0.035), lightgreen module (MDC = 0.641, *p* = 0.026), magenta module (MDC = 0.649, *p* = 0.002), and red (MDC = 0.703, *p* = 0.002).

Compared with HC, MDD_CM patients presented a loss of connectivity in 6 out of the 27 modules (22.2%), whereas 21 modules (77.8%) presented no changes in network connectivity; MDD_nCM patients presented decreased connectivity in 1 module (3.7%) and increased connectivity in 1 module (3.7%), with 25 modules (92.6%) showing no change in network connectivity. Compared with MDD_nCM, MDD_CM showed decreased connectivity in two modules (7.2%) and increased connectivity in 1 module (3.7%), with 25 modules (89.1%) showing no change in network connectivity.

### 3.4. Functional Annotation of the Co-Expression Modules

**Figure 4:**
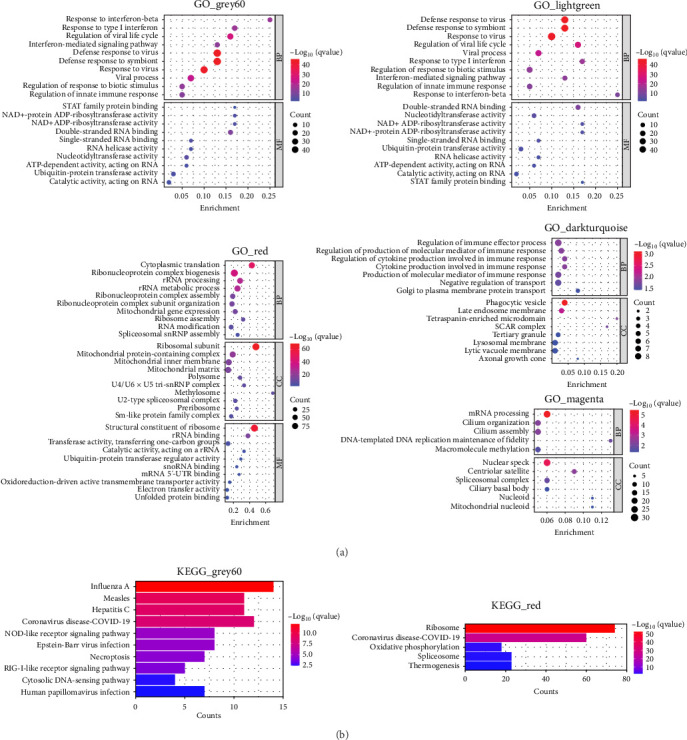
Significant functional terms of genes in the modules. (A) Bubble chart displaying the significant GO terms of grey60, red, lightgreen, darkurquoise, and magenta modules. (B) The bar chart shows the KEGG terms of grey60 and red modules. BP, biological process; CC, cell component; GO, gene ontology; KEGG, Kyoto Encyclopedia of Genes and Genomes; MF, molecular function..

**Figure 5:**
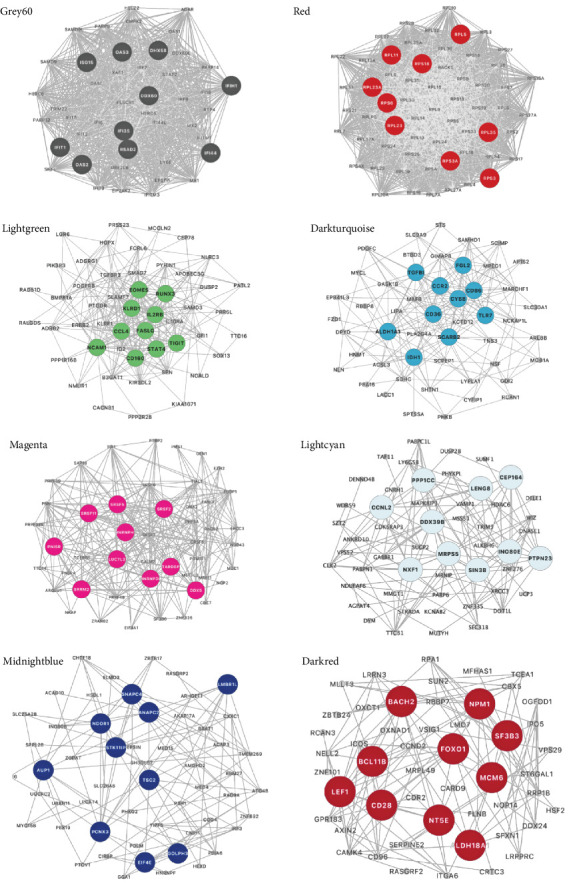
The protein–protein interaction network and hub genes in the modules. These networks highlight top 10 hub genes with the highest degree of connectivity in the protein–protein interaction network of eight differential modules across the three groups.

We performed GO and KEGG enrichment analyses to associate modules with biological pathways and functions. The grey60 module was linked to several pathways closely related to immune response and virus infection, including the type 1 interferon signaling pathway, regulation of innate immune response, and NOD-like receptor signaling pathway. The hub genes in this module are part of the body's defense mechanism against viral infections and are typically induced by interferons. The red module was enriched primarily in biosynthetic and metabolic processes, such as cytoplasmic translation, rRNA metabolic processing, mitochondrial gene expression, and thermogenesis, with the hub genes encoding mainly various ribosomal proteins. The lightgreen module was mainly associated with the negative regulation of immune system processes, including T-cell activation and lymphocyte differentiation, phosphatidylinositol and inositol lipid-mediated signaling, with hub genes playing an essential role in immune function. The darkturquoise module was linked to the regulation of the production of molecular mediators of immune response, with the hub genes involved mainly in immune function. The magenta module was mainly associated with mRNA processing (Figures 4 and 5).

The remaining three modules (greencyan, midnightblue, and darkred) did not show significant results in the enrichment analysis, since no *p*-values survived after multiple tests were corrected. The hub genes in the darkred module were mainly involved in immune function, metabolism, and transcription regulation, whereas the hub genes in greencyan and midnightblue were involved mainly in physiological processes (Figure 5).

### 3.5. Correlation Between the Co-Expression Modules and FC Brain Network and Depressive Symptoms

**Figure 6:**
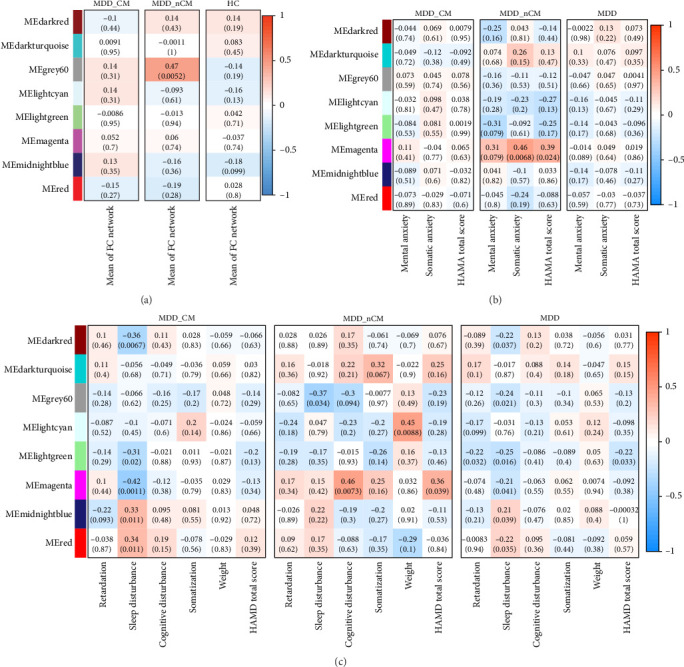
Correlations between gene co-expression modules and FC or depressive symptoms. (A) Correlations between gene co-expression modules and mean of FC network in MDD_CM, MDD_nCM, or HC groups. (B) Correlations between gene co-expression modules and HAMA scores in MDD_CM, MDD_nCM, or MDD groups. (C) Correlations between gene co-expression modules and HAMD scores in MDD_CM, MDD_nCM, or MDD groups. HC, healthy control; FC, functional connectivity; HAMA, Hamilton anxiety rating scale; HAMD, Hamilton depression rating scale; HC, healthy control; MDD_CM, major depressive disorder with childhood maltreatment; MDD_nCM, major depressive disorder without childhood maltreatment. The values in each block indicate the corresponding *r* and *p* values of the correlation analysis.

In the analysis of the correlation between the average FC of all edges in the brain network and the co-expression modules, the results revealed a significantly positive correlation between the gray60 module and the average FC in the MDD_nCM group (*r* = −0.47, *p* = 0.0052) (Figure 6A). Analysis of the correlations between co-expression modules and depressive symptoms revealed that sleep disturbance was associated with red module (*r* = 0.34, *p* = 0.011), darkred module (*r* = −0.36, *p* = 0.0067), lightgreen module (*r* = −0.31, *p* = 0.02), magenta module (*r* = −0.42, *p* = 0.0011), and midnightblue module (*r* = 0.33, *p* = 0.011) in the MDD_CM group, and that the magenta module was associated with cognitive disturbance (*r* = 0.46, *p* = 0.0073) and somatic anxiety (*r* = 0.46, *p* = 0.0068) in the MDD_nCM group (Figure 6B,C). The comparison of correlation coefficients between the MDD_CM and MDD_nCM groups showed significant differences in the relationships between magenta and sleep disturbance (*FDR-p* = 0.045), as well as between magenta and cognitive disturbance (*FDR-p* = 0.034) (see Table S6).

## 4. Discussion

This study used data-driven approaches to identify differences in brain functional connectivity and blood transcriptomics among the MDD_CM, MDD_nCM, and HC groups. Specifically, an IOG-centered functional connectivity network, with key regions primarily located in the visual network, DMN, ECN, limbic system, and cerebellum, showed significant variations among the three groups. Additionally, differences in the transcriptomic profiles related to immune and metabolic processes were observed between the MDD_CM and MDD_nCM groups. These findings enhance our understanding of the biological mechanisms of MDD_CM.

Our results revealed that both MDD and CM were associated with significant alterations in the IOG-centered functional connectivity network. Moreover, individuals with MDD and a history of CM exhibited even lower connectivity in this functional network than did those with MDD but without CM. This finding is consistent with a large body of prior research showing that MDD patients with CM are associated with more widespread alterations of brain structure and function [5, 32, 33]. Studies also suggest that different mental disorders, or even varying symptoms of the same disorder, may correspond to distinct brain network changes [34, 35], although shared neural networks across different symptoms are also possible [36]. Our findings emphasize that reduced connectivity, particularly involving regions in the DMN, ECN, visual network, limbic system, and cerebellum, may present common neuroimaging mechanisms underlying both CM and MDD.

One key finding in the brain network is the involvement of the MOG and IOG, which are core regions of the visual network. Prior studies have shown that these regions are typically involved in visual processing, and abnormal neural activity in the MOG and IOG has been previously observed in patients with MDD [32, 37, 38]. Our study extends these findings by showing that individuals with MDD and a history of CM exhibit lower connectivity in the visual network than those without CM. This may suggest that early life adversity not only affects regions directly involved in emotional and cognitive regulation but also alters sensory processing systems, contributing to the attentional and perceptual biases commonly observed in MDD patients, particularly toward negative stimuli [39].

The other core brain regions are located mainly in the CEN and DMN networks. Notably, key regions in the DMN, including the SFGmed and OFCmed, showed altered connectivity, aligning with their roles in self-referential thinking and emotional regulation. The OFCmed, in particular, is critical for reward processing. Its heightened sensitivity to nonreward and punishment signals may contribute to the persistent negative cognitive patterns observed in depression, potentially exacerbated by early life stressors [40]. Dysfunction in the DMN, commonly observed in patients with MDD, is often associated with excessive rumination and impaired emotional regulation, both of which are further intensified by CM. The ECN, involving regions such as PFCventmed and rectus gyrus, also showed significant connectivity disruptions. The ECN plays a pivotal role in cognitive control and attention regulation, which are essential for adaptive emotional responses. Damage or dysregulation in the PFC and OFC has been linked to difficulties in modulating fear and anxiety, as well as diminished executive control, which further impairs emotion regulation [41]. In line with these insights, our research supports a prior study indicating impaired FC between the OFC and PFC in MDD patients who have experienced CM [32]. These findings suggest that CM compounds the typical functional disruptions in the DMN and ECN observed in MDD, contributing to more severe emotional dysregulation and cognitive impairments [42]. These disruptions in emotional regulation and self-referential thinking may underlie the long-lasting effects of CM on brain function, increasing vulnerability to depression later in life.

Our study revealed significant alterations in cerebellar regions, particularly lobules IV, V (CER4_5), and VI (CER_6), which were associated with CM. These findings emphasize the role of the cerebellum in emotional processing beyond its traditional function in motor control. Traumatic stress may disrupt the cerebellum-based predictive system, leading to overestimation of negative outcomes and inappropriate responses to stress [43]. Specific cerebellar regions, such as those involved in sensorimotor control (CER4_5), autobiographical memory, working memory, and attention (CER6_7), are implicated in trauma-related processes [44, 45]. Reduced connectivity between the cerebellum and occipital lobe, a finding supported by previous research linking it to depression [46], further highlights the cerebellum's role in integrating sensory and emotional information. Disruptions in this connectivity may contribute to the emotional dysregulation in individuals with both MDD and a history of CM.

Our transcriptomics analysis revealed an association between CM and the immune response, specifically the type 1 interferon signaling pathway. These findings support the link between CM and the dysregulation of interferon-mediated signaling pathways [16] and inflammatory signaling pathways [9, 13, 47–49]. The CM-related immune phenotype is characterized by impairment of innate immunity, inflammation, and the cellular immune system [50]. Individuals with CM presented upregulation of the upstream signaling of proinflammatory factors during acute stress, which was also consistent with our results [51]. Moreover, compared with healthy individuals without CM, MDD patients with a history of CM presented with dysregulated T-cell activation, lymphocyte differentiation, and molecular mediators of the immune response, as well as phosphatidylinositol and inositol lipid-mediated signaling. These finding indicate that CM results in complex inflammatory immune system changes in patients with MDD. Our findings indicate that transcriptional profiles promote chronic low-grade inflammation during traumatic stress, thereby providing a mechanistic link between stress and the development of inflammation-related diseases [52, 53]. MDD, on the other hand, is considered as a chronic, low-grade state of inflammation, even when not infected by bacteria [54]. Inflammation has also been proposed to mediate the relationship between CM and depression [55]. Hence, dysregulation of the transcriptome of immune-inflammatory pathways represents a crucial link between CM and MDD.

There is a correlation between the abnormal connectivity of brain networks and immune-related gene modules, suggesting that the abnormal brain networks in MDD patients may be regulated by immune-related pathways. Consistent with our findings, previous studies have shown that dysconnectivity of a brain functional network is associated with peripheral inflammation in MDD patients [56, 57]. Peripheral administration of IFN-*α* quickly reduces the efficiency and node degree of the temporo-occipital network, which is directly linked to mood impairments [58]. Relationships between peripheral inflammation and rsFC within an emotion regulation network and the central executive network have also been reported [59]. Dysregulation of immune pathway transcription and changes in the IOG-centered brain network changes are potential biological mechanisms related to CM and MDD [60].

The findings of the present study support that immunity is not associated with MDD as a single syndrome, but rather with the presence and progression of specific depressive symptoms [61]. Previous studies have shown that immune status is specifically related to changes in sleep disturbance, tiredness or low energy, and appetite [62–64]. These symptoms indicate sickness behaviors observed in physically ill individuals. Therefore, somatic symptoms of MDD may be a manifestation of a specific immunological endophenotype of MDD [65]. This finding aligns with the evolutionary perspective that links immunity and MDD to host defense against pathogens [66]. Our results provide transcriptomic clues into symptom-specific immune abnormalities in patients with MDD, particularly those with childhood traumatic experiences.

We need to acknowledge some limitations of this study. First, the classification of CM relied on participants' self-reported data, and given the cross-sectional nature of the study, causality could not be established. Second, the interpretation of gene modules within the field of transcriptomics poses intricate challenges, necessitating further research to examine targeted gene expression levels for more precise findings. Third, our correlation analysis was not adjusted for multiple comparison corrections, and the results should be interpreted as exploratory. Fourth, the limited variance in CM scores in the patient group without trauma may compromise statistical power. Finally, the adverse impacts of CM were evident in patients without a history of such maltreatment. We suggest including a group of healthy individuals who have a history of CM but do not have MDD in future studies.

## 5. Conclusions

In conclusion, there is a notable relationship between CM and depression, both of which are associated with decreased FC in the visual, default mode, and central executive networks. Furthermore, blood transcriptomic profiles related to immune and metabolic processes have been linked to CM in individuals with depression. These findings regarding the brain network and immune gene architectural profiles associated with CM and depression contribute significantly to our understanding of the pathophysiology of depression with a history of CM.

## Figures and Tables

**Table 1:** Demographics and clinical characteristics of all participants.

Terms	MDD_CM (*n* = 78)	MDD_nCM (*n* = 61)	HC (*n* = 126)	*df*	*Statistics*	*p*
Age (year)	25.68 (8.55)	27.7 (9.73)	26.07 (8.97)	264	0.961^a^	0.384
Sex (male/female)	26/52	22/39	45/81	2	0.153^b^	0.926
Years of education	13.46 (2.8)	13.93 (2.9)	14.37 (2.71)	264	2.56^a^	0.079
Total disease duration (months)	42.37 (59.2)	29.9 (46.68)	NA	137	1.76^c^	0.186
Number of episodes	2.3 (3.04)	1.68 (1.38)	NA	137	2.03^c^	0.157
HAMD total score	21 (7)	21 (7)	NA	NA	−0.0234^d^	0.981
Retardation	7 (2)	7 (3)	NA	NA	−0.0408^d^	0.967
Sleep disturbance	3.5 (2.75)	3 (3)	NA	NA	−0.688^d^	0.492
Cognitive disturbance	4 (2)	4 (3)	NA	NA	−0.028^d^	0.978
Somatization	4 (2)	4 (2)	NA	NA	−0.432^d^	0.665
Weight	1 (2)	1 (2)	NA	NA	−0.876^d^	0.381
HAMA total score	14 (9)	16 (10)	NA	NA	−1.31^d^	0.191
Mental anxiety	11 (5)	11 (6)	NA	NA	−0.677^d^	0.498
Somatic anxiety	3 (5)	5 (6)	NA	NA	−1.5^d^	0.134
FD (mm)	0.08 (0.03)	0.07 (0.02)	0.07 (0.03)	264	0.51^a^	0.601
CTQ, total score	52.3 (11.2)	32 (4.7)	30 (4)	264	262^a^	<0.001
Emotional abuse	11.4 (4.7)	6.6 (1.9)	6 (1.3)	264	91.9^a^	<0.001
Physical abuse	7.7 (3.5)	5.5 (1)	5.5 (1)	264	30.5^a^	<0.001
Sexual abuse	6 (1.8)	5.1 (0.4)	5.1 (0.4)	264	18^a^	<0.001
Emotional neglect	16.1 (4)	8.7 (2.8)	7.5 (2.3)	264	209^a^	<0.001
Physical neglect	11.2 (3.1)	6 (1.2)	5.9 (1.3)	264	195^a^	<0.001

Abbreviations: CTQ, Childhood Trauma Questionnaire; df, degrees of freedom; FD, framewise displacement; HAMA, Hamilton Anxiety Scale; HAMD, Hamilton Depression Scale; HC, healthy control; MDD_CM, major depressive disorder with childhood maltreatment; MDD_nCM, major depressive disorder without childhood maltreatment.

^a^F statistics.

^b^c^2^ statistics.

^c^t statistics.

^d^Wilcoxon test.

**Table 2:** Comparison of module differential connectivity among groups.

Module	MDC value	*p*	Comparison
Red	0.726	0.022	MDD_CM *vs*. MDD_nCM
	0.703	0.002	MDD_CM *vs*. HC

Grey60	0.700	0.035	MDD_CM *vs*. MDD_nCM
	1.383	0.043	MDD_nCM *vs*. HC

Midnightblue	1.203	0.019	MDD_CM *vs*. MDD_nCM
	0.794	0.014	MDD_nCM *vs*. HC

Darkred	0.792	0.019	MDD_CM *vs*. HC

Darkturquoise	0.645	0.035	MDD_CM *vs*. HC

Lightcyan	0.713	0.035	MDD_CM *vs*. HC

Lightgreen	0.641	0.026	MDD_CM *vs*. HC

Magenta	0.649	0.002	MDD_CM *vs*. HC

Abbreviations: HC, healthy control; MDC, module differential connectivity; MDD_CM, major depressive disorder with childhood maltreatment; MDD_nCM, major depressive disorder without childhood maltreatment.

## Data Availability

The raw data supporting this study's findings are available from the corresponding author.

## Supporting Information

Supporting Information Figure S1. Participant enrollment and quality control workflow. Figure S2. Gene co-expression module analysis. (A) Analysis of gene expression network topology for various soft-thresholding powers. (B) Genes co-expression module clustering dendrograms. Table S1. Demographics and clinical measures of the participants involved in transcriptome analysis. Table S2. Comparison of the brain network among the three groups. Table S3. Regression analysis of the differential brain network and continuous variables of childhood trauma in the MDD group. Table S4. Size of the co-expressed gene module. Table S5. Comparison of module preservation among MDD_CM, MDD_nCM, and HC groups. Table S6. Comparison of models-symptom correlations between MDD_CM and MDD_nCM groups (*FDR-p*).
