# Supplementary material for: Altered Brain Functional Connectivity and Peripheral Transcriptomic Profiles in Major Depressive Disorder With Childhood Maltreatment
Source: Depress Anxiety. 2025 Mar 31;2025:6059502. doi: 10.1155/da/6059502 (PMC11976050; doi:10.1155/da/6059502)
Supplement: Supporting Information — Figure S1. Participant enrollment and quality control workflow. Figure S2. Gene co-expression module analysis. (A) Analysis of gene expression network topology for various soft-thresholding powers. (B) Genes co-expression module clustering dendrograms. Table S1. Demographics and clinical measures of the participants involved in transcriptome analysis. Table S2. Comparison of the brain network among the three groups. Table S3. Regression analysis of the differential brain network and continuous variables of childhood trauma in the MDD group. Table S4. Size of the co-expressed gene module. Table S5. Comparison of module preservation among MDD_CM, MDD_nCM, and HC groups. Table S6. Comparison of models-symptom correlations between MDD_CM and MDD_nCM groups (FDR-p). [file 6059502.f1.docx]

**Supplementary information**

**Altered Brain Functional Connectivity and Peripheral Transcriptomic Profiles in Major Depressive Disorder with Childhood Maltreatment**

Min Wang^1#^, Jinxue Wei^1#^, Yushun Yan^1^, Yue Du^1^, Huanhuan Fan^1^, Yikai Dou^1^, Liansheng Zhao^1^, Rongjun Ni^1^, Xiao Yang^1*^, Xiaohong Ma^1*^

^1^ Mental Health Center and Institute of Psychiatry, West China Hospital of Sichuan University, Chengdu, Sichuan, China.

**Table S1.** Demographics and clinical measures of the participants involved in transcriptome analysis.

**Table S2.** Comparison of the brain network among the three groups.

**Table S3.** Regression analysis of the differential brain network and continuous variables of childhood trauma in the MDD group.

**Table S4.** Size of the co-expressed gene module.

**Table S5.** Comparison of module preservation among MDD_CM, MDD_nCM, and HC groups.

**Table S6.** Comparison of models-symptom correlations between MDD_CM and MDD_nCM groups (*FDR-p*).

**Figure S1** Participant enrollment and quality control workflow.

**Figure S2 Gene co-expression module analysis.** (A) Analysis of gene expression network topology for various soft-thresholding powers. (B) Genes co-expression module clustering dendrograms.

**Methods
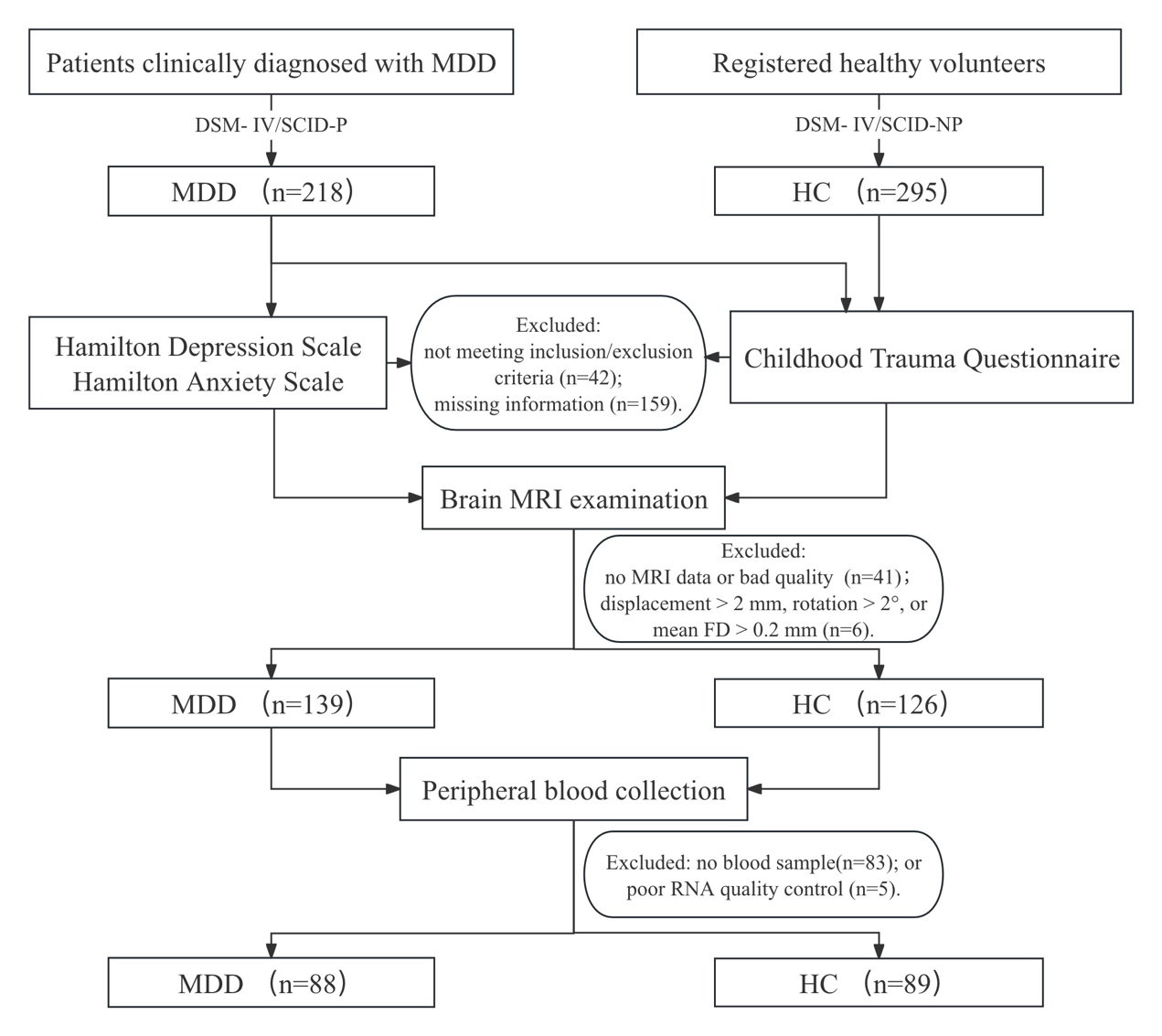
**

**Figure S1** Participant enrollment and quality control workflow.

DSM- IV, diagnostic and statistical manual of mental disorders, fourth edition; DSM-IV/SCID-P, structured clinical interview for DSM-IV - patient version; DSM-IV/SCID-NP, structured clinical interview for DSM-IV - non-patient version; MDD, major depressive disorder; HC, healthy controls; MRI, magnetic resonance imaging; FD, framewise displacement.

**Results**

**Table S1.** Demographics and clinical measures of the participants involved in transcriptome analysis.

| Terms | MDD_CM (n=51) | MDD_nCM (n=37) | HC (n=89) | *df* | *Statistics* | *p* |
| --- | --- | --- | --- | --- | --- | --- |
| Age (year) | 24.7 (7.14) | 27.16 (9.39) | 24.42 (6.5) | 176 | 1.92^a^ | 0.149 |
| Sex (male/female) | 17/44 | 14/23 | 28/61 | 2 | 1.06^b^ | 0.588 |
| Years of education | 13.48 (2.74) | 13.78 (2.74) | 14.37 (2.66) | 176 | 2.08 ^a^ | 0.127 |
| Total disease duration (months) | 34.42 (46.4) | 25.46 (31.97) | NA | 176 | 1.02^c^ | 0.316 |
| Number of Episodes | 2.02 (2.73) | 1.72 (1.65) | NA | 176 | 0.316 ^c^ | 0.575 |
| HAMD total score | 21 (6) | 21 (7) | NA | NA | -0.36 ^d^ | 0.719 |
| Retardation | 7 (2) | 8 (3) | NA | NA | -0.787 ^d^ | 0.431 |
| Sleep disturbance | 3 (3) | 4 (3) | NA | NA | -0.123 ^d^ | 0.902 |
| Cognitive disturbance | 4 (2) | 4 (2) | NA | NA | -0.504 ^d^ | 0.614 |
| Somatization | 4 (2) | 4 (2) | NA | NA | -0.405 ^d^ | 0.685 |
| Weight | 1 (2) | 1 (2) | NA | NA | -0.879 ^d^ | 0.379 |
| HAMA total score | 14 (9) | 20 (10) | NA | NA | -2.28 ^d^ | 0.023 |
| Mental anxiety | 11 (5) | 13 (7) | NA | NA | -1.74 ^d^ | 0.0818 |
| Somatic anxiety | 3 (5) | 6 (5) | NA | NA | -2.12 ^d^ | 0.0342 |
| FD (mm) | 0.08 (0.03) | 0.07 (0.02) | 0.07 (0.03) | 176 | 0.785 ^a^ | 0.458 |
| CTQ, total score | 51.9 (11.1) | 32 (4.5) | 30 (4) | 176 | 183 ^a^ | <0.001 |
| Emotional abuse | 11.2 (4.4) | 6.3 (2) | 6 (1.3) | 176 | 69.8 ^a^ | <0.001 |
| Physical abuse | 7.3 (3.2) | 5.6 (1.2) | 5.5 (1) | 176 | 14.7 ^a^ | <0.001 |
| Sexual abuse | 6.1 (2) | 5.1 (0.3) | 5.1 (0.4) | 176 | 15.6 ^a^ | <0.001 |
| Emotional neglect | 16 (4) | 8.9 (2.7) | 7.4 (2.3) | 176 | 155 ^a^ | <0.001 |
| Physical neglect | 11.4 (3.1) | 6 (1.2) | 5.9 (1.3) | 176 | 141 ^a^ | <0.001 |

MDD_CM: major depressive disorder with childhood maltreatment; MDD_nCM: major depressive disorder without childhood maltreatment; HC: healthy control; df, degrees of freedom; HAMD, Hamilton Depression Scale; HAMA, Hamilton Anxiety Scale; FD, framewise displacement; CTQ, Childhood Trauma Questionnaire.

a, F statistics; b, c^2^ statistics; c, t statistics; d, Wilcoxon test.

**Table S2.** Comparison of the brain network among the three groups.

| Plot | Boxplot | | Density | |
| --- | --- | --- | --- | --- |
| Comparison | *t* | *p* | *D* | *p* |
| MDD_CM vs. MDD_nCM | 2.208 | 0.029 | 0.313 | 0.001 |
| MDD_CM vs. HC | 5.917 | <0.001 | 0.458 | <0.001 |
| MDD_nCM vs. HC | 3.165 | 0.002 | 0.313 | 0.001 |

MDD_CM, major depressive disorder with childhood maltreatment; MDD_nCM, major depressive disorder without childhood maltreatment; HC, healthy control.

**Table S3.** Regression analysis of the differential brain network and continuous variables of childhood trauma in the MDD group.

| Variables | Estimate | *t* | *p* |
| --- | --- | --- | --- |
| Emotional abuse | -0.005 | -2.375 | 0.018 |
| Emotional neglect | -0.006 | -3.666 | <0.001 |
| Physical abuse | -0.006 | -1.582 | 0.115 |
| Physical neglect | -0.008 | -3.073 | 0.002 |
| Sexual abuse | -0.015 | -2.038 | 0.043 |
| Total scores of CTQ | -0.002 | -3.467 | 0.001 |

CTQ, childhood trauma questionnaire.


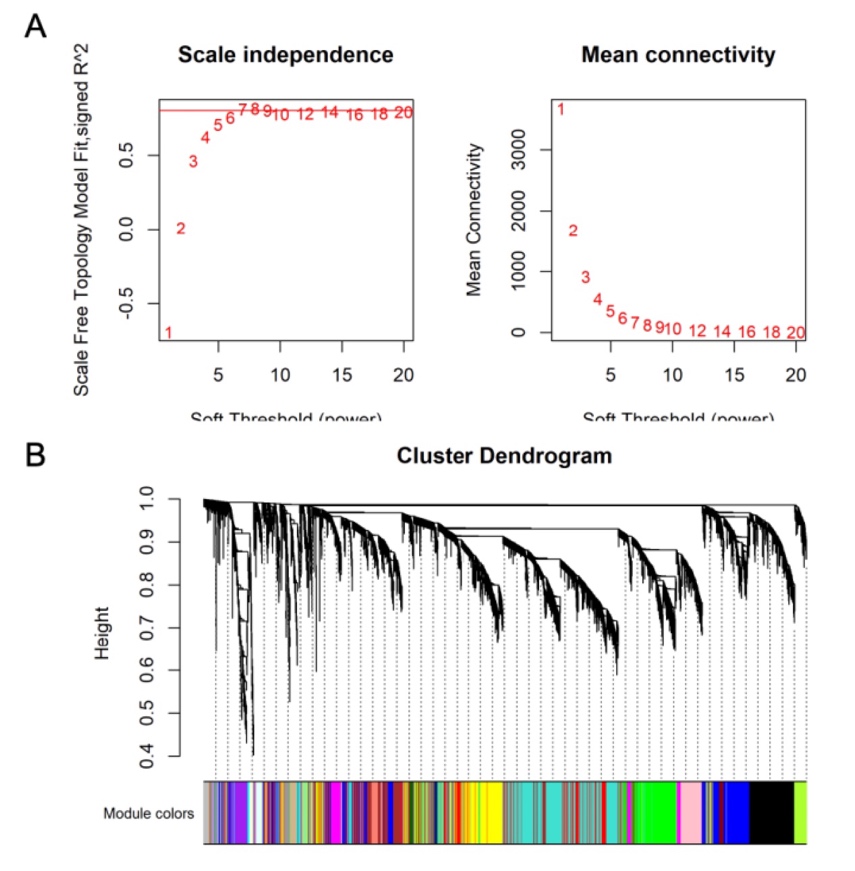


**Figure S2. Gene co-expression modules analysis.** (A) Analysis of gene expression network topology for various soft-thresholding powers. (B) Genes co-expression module clustering dendrograms.

**Table S4.** Size of the co-expressed gene module.

| Module | Size |  | Module | Size |
| --- | --- | --- | --- | --- |
| black | 874 |  | grey | 296 |
| blue | 1273 |  | grey60 | 96 |
| brown | 1158 |  | lightcyan | 106 |
| cyan | 110 |  | lightgreen | 89 |
| darkgreen | 78 |  | lightyellow | 85 |
| darkgrey | 49 |  | magenta | 399 |
| darkorange | 36 |  | midnightblue | 106 |
| darkred | 83 |  | orange | 38 |
| darkturquoise | 66 |  | pink | 571 |
| gold | 1000 |  | purple | 299 |
| green | 989 |  | red | 926 |
| greenyellow | 228 |  | royalblue | 83 |
| turquoise | 2260 |  | salmon | 143 |
| yellow | 1108 |  | tan | 159 |

**Table S5** Comparison of module preservation among MDD_CM, MDD_nCM, and HC groups.

| Module | **Z_summary_** |  | **density** | | | | |  | **connectivity** | | | | | |
| --- | --- | --- | --- | --- | --- | --- | --- | --- | --- | --- | --- | --- | --- | --- |
|  |  |  | **Z_density_** | **Z_propVar_**  **_Explained_** | **Z._meanSign_**  **_AwareKME_** | **Z._meanSign_**  **_AwareCorDat_** | **Z._meanAdj_** |  | **Z_connectivity_** | | | **Z._cor.kIM_** | **Z._cor.kME_** | **Z._cor.cor_** |
| **MDD_CM vs. MDD_nCM** | | | | | | | | | | | | | | |
| black | 33.445 |  | 20.874 | -1.425 | 43.173 | 431.563 | -6.703 |  | 46.016 | 25.243 | | | 46.016 | 482.928 |
| blue | 46.791 |  | 35.448 | 8.743 | 62.153 | 909.995 | 3.978 |  | 58.135 | 30.857 | | | 58.135 | 801.541 |
| brown | 51.539 |  | 49.161 | 25.653 | 65.964 | 1068.280 | 32.359 |  | 53.916 | 29.025 | | | 53.916 | 765.558 |
| cyan | 20.577 |  | 25.217 | 12.068 | 20.861 | 106.147 | 29.574 |  | 15.937 | 7.821 | | | 15.937 | 67.516 |
| darkgreen | 9.847 |  | 6.812 | 0.570 | 13.054 | 47.054 | -1.484 |  | 12.882 | 5.642 | | | 12.882 | 47.877 |
| darkgrey | 7.211 |  | 9.367 | 4.758 | 12.183 | 42.160 | 6.552 |  | 5.055 | 5.055 | | | 4.752 | 17.471 |
| darkorange | 7.417 |  | 10.092 | 5.791 | 11.554 | 37.905 | 8.630 |  | 4.743 | 4.743 | | | 4.544 | 12.885 |
| darkred | 12.831 |  | 11.856 | 7.225 | 16.488 | 70.675 | 7.126 |  | 13.805 | 6.531 | | | 13.805 | 50.125 |
| darkturquoise | 8.371 |  | 8.451 | 3.619 | 13.284 | 46.664 | 2.392 |  | 8.291 | 5.552 | | | 8.291 | 29.739 |
| gold | 34.613 |  | 21.702 | 0.810 | 41.994 | 507.962 | 1.410 |  | 47.524 | 26.004 | | | 47.524 | 597.412 |
| green | 53.735 |  | 57.191 | 29.929 | 53.955 | 488.904 | 60.427 |  | 50.279 | 29.618 | | | 50.279 | 385.666 |
| greenyellow | 20.933 |  | 17.805 | 8.718 | 26.892 | 165.915 | 7.193 |  | 24.060 | 12.427 | | | 24.060 | 130.163 |
| grey60 | 10.978 |  | 9.874 | 1.838 | 14.226 | 53.516 | 5.521 |  | 12.083 | 6.517 | | | 12.083 | 45.718 |
| lightcyan | 11.187 |  | 8.160 | 1.429 | 14.891 | 56.447 | -1.267 |  | 14.213 | 8.007 | | | 14.213 | 55.347 |
| lightgreen | 7.952 |  | 6.402 | 0.024 | 12.780 | 42.423 | -1.822 |  | 9.502 | 7.248 | | | 9.502 | 36.427 |
| lightyellow | 3.280 |  | 1.557 | -4.750 | 5.853 | 14.231 | -2.739 |  | 5.003 | 5.003 | | | 4.620 | 20.045 |
| magenta | 25.275 |  | 21.105 | 10.138 | 32.071 | 204.723 | 9.883 |  | 29.445 | 16.721 | | | 29.445 | 188.577 |
| midnightblue | 18.043 |  | 20.631 | 10.466 | 19.653 | 98.126 | 21.608 |  | 15.456 | 8.189 | | | 15.456 | 65.017 |
| orange | 9.242 |  | 11.628 | 6.435 | 11.834 | 39.184 | 11.422 |  | 6.857 | 5.288 | | | 6.857 | 18.262 |
| pink | 45.853 |  | 55.339 | 27.678 | 47.385 | 380.956 | 63.292 |  | 36.367 | 21.468 | | | 36.367 | 252.821 |
| purple | 18.905 |  | 18.493 | 5.644 | 25.218 | 131.996 | 11.768 |  | 19.318 | 12.555 | | | 19.318 | 110.981 |
| red | 34.908 |  | 23.802 | 4.362 | 43.242 | 349.440 | 2.330 |  | 46.013 | 26.634 | | | 46.013 | 396.471 |
| royalblue | 11.498 |  | 12.895 | 6.642 | 15.533 | 62.403 | 10.256 |  | 10.101 | 7.973 | | | 10.101 | 39.307 |
| salmon | 22.198 |  | 26.410 | 14.161 | 24.206 | 149.542 | 28.613 |  | 17.987 | 10.028 | | | 17.987 | 87.901 |
| tan | 7.370 |  | 3.209 | -8.011 | 10.984 | 31.070 | -4.566 |  | 11.532 | 6.928 | | | 11.532 | 51.602 |
| turquoise | 76.175 |  | 76.162 | 42.763 | 84.548 | 1166.176 | 67.777 |  | 76.187 | 40.146 | | | 76.187 | 869.876 |
| yellow | 42.820 |  | 34.038 | 13.514 | 50.904 | 503.123 | 17.171 |  | 51.603 | 30.655 | | | 51.603 | 465.367 |
| **MDD_CM vs. HC** | | | | | | | | | | | | | | |
| black | 41.707 |  | 34.642 | 12.661 | 56.624 | 712.382 | 8.462 |  | 48.771 | | 27.161 | | 48.771 | 613.367 |
| blue | 48.285 |  | 37.617 | 11.727 | 63.507 | 913.915 | 4.886 |  | 58.953 | | 32.772 | | 58.953 | 834.442 |
| brown | 50.184 |  | 45.583 | 23.842 | 67.324 | 1092.311 | 23.776 |  | 54.785 | | 33.425 | | 54.785 | 828.748 |
| cyan | 18.497 |  | 21.644 | 11.342 | 21.339 | 113.503 | 21.949 |  | 15.350 | | 8.912 | | 15.350 | 70.554 |
| darkgreen | 10.852 |  | 9.109 | 3.770 | 14.448 | 57.440 | 0.689 |  | 12.596 | | 7.212 | | 12.596 | 50.019 |
| darkgrey | 9.475 |  | 11.830 | 6.607 | 13.071 | 48.204 | 10.588 |  | 7.121 | | 5.146 | | 7.121 | 21.003 |
| darkorange | 7.669 |  | 8.885 | 5.762 | 10.909 | 34.596 | 6.860 |  | 6.453 | | 5.322 | | 6.453 | 17.988 |
| darkred | 12.516 |  | 12.207 | 7.459 | 16.956 | 78.893 | 5.505 |  | 12.824 | | 6.138 | | 12.824 | 47.417 |
| darkturquoise | 8.640 |  | 8.123 | 3.308 | 12.938 | 48.642 | 0.888 |  | 9.156 | | 6.476 | | 9.156 | 31.248 |
| gold | 33.984 |  | 20.337 | -0.640 | 41.314 | 503.978 | -1.227 |  | 47.631 | | 28.142 | | 47.631 | 630.497 |
| green | 49.150 |  | 52.265 | 26.656 | 59.483 | 631.986 | 45.047 |  | 46.034 | | 28.073 | | 46.034 | 492.780 |
| greenyellow | 20.918 |  | 18.103 | 8.938 | 27.267 | 179.569 | 5.574 |  | 23.734 | | 12.655 | | 23.734 | 145.618 |
| grey60 | 20.512 |  | 28.638 | 12.541 | 21.597 | 118.361 | 35.678 |  | 12.387 | | 6.967 | | 12.387 | 53.489 |
| lightcyan | 12.218 |  | 9.694 | 3.156 | 16.232 | 71.572 | -0.247 |  | 14.742 | | 8.587 | | 14.742 | 60.667 |
| lightgreen | 9.093 |  | 9.194 | 2.775 | 15.613 | 63.484 | 0.484 |  | 8.992 | | 6.782 | | 8.992 | 38.359 |
| lightyellow | 36.777 |  | 63.337 | 16.373 | 21.470 | 112.805 | 105.204 |  | 10.217 | | 7.037 | | 10.217 | 44.367 |
| magenta | 24.711 |  | 19.805 | 8.139 | 31.471 | 236.982 | 3.678 |  | 29.617 | | 16.879 | | 29.617 | 223.980 |
| midnightblue | 17.137 |  | 18.609 | 9.810 | 20.726 | 110.110 | 16.491 |  | 15.666 | | 9.224 | | 15.666 | 73.816 |
| orange | 9.571 |  | 11.855 | 6.729 | 11.685 | 39.933 | 12.025 |  | 7.286 | | 5.646 | | 7.286 | 19.813 |
| pink | 41.822 |  | 48.747 | 25.502 | 50.823 | 495.181 | 46.672 |  | 34.896 | | 20.371 | | 34.896 | 307.245 |
| purple | 32.706 |  | 42.411 | 16.162 | 33.378 | 242.878 | 51.444 |  | 23.001 | | 12.924 | | 23.001 | 159.399 |
| red | 40.654 |  | 35.542 | 14.427 | 56.656 | 608.774 | 14.178 |  | 45.766 | | 28.371 | | 45.766 | 543.350 |
| royalblue | 17.064 |  | 23.695 | 12.031 | 19.012 | 97.008 | 28.378 |  | 10.433 | | 7.682 | | 10.433 | 40.473 |
| salmon | 23.428 |  | 27.335 | 15.073 | 27.485 | 186.945 | 27.185 |  | 19.520 | | 10.237 | | 19.520 | 104.383 |
| tan | 9.992 |  | 7.132 | -2.028 | 16.293 | 64.895 | -2.177 |  | 12.851 | | 7.881 | | 12.851 | 70.204 |
| turquoise | 83.553 |  | 90.513 | 48.872 | 97.750 | 1805.070 | 83.276 |  | 76.592 | | 44.061 | | 76.592 | 1251.051 |
| yellow | 49.816 |  | 46.142 | 20.686 | 61.134 | 790.349 | 31.149 |  | 53.490 | | 30.891 | | 53.490 | 660.527 |

propVarExplained, proportion of variance explained; meanSignAwareKME, mean sign-aware module membership; meanSignAwareCorDat, mean sign-aware correlation; meanAdj, mean adjacency; cor.kIM, correlation of intramodular connectivities; cor.kME, correlation of module membership; cor.cor, correlation of correlation coefficients.

**Table S6.** Comparison of models-symptom correlations between MDD_CM and MDD_nCM groups (*FDR-p*).

| **Items** | **Brain network** | **Mental anxiety** | **Somatic anxiety** | **HAMA total score** | **Retardation** | **Sleep disturbance** | **Cognitive disturbance** | **Somatization** | **Weight** | **HAMD Total Score** |
| --- | --- | --- | --- | --- | --- | --- | --- | --- | --- | --- |
| MEdarkred | 0.556 | 0.651 | 0.900 | 0.653 | 0.810 | 0.248 | 0.891 | 0.680 | 0.990 | 0.720 |
| MEdarkturquoise | 0.970 | 0.651 | 0.292 | 0.576 | 0.810 | 0.860 | 0.420 | 0.320 | 0.990 | 0.720 |
| MEgrey60 | 0.556 | 0.651 | 0.537 | 0.576 | 0.810 | 0.373 | 0.653 | 0.600 | 0.990 | 0.740 |
| MElightcyan | 0.556 | 0.651 | 0.320 | 0.560 | 0.810 | 0.663 | 0.653 | 0.320 | 0.152 | 0.720 |
| MElightgreen | 0.970 | 0.651 | 0.537 | 0.576 | 0.810 | 0.663 | 0.980 | 0.320 | 0.990 | 0.740 |
| MEmagenta | 0.970 | 0.651 | 0.104 | 0.560 | 0.810 | **0.045** | **0.034** | 0.320 | 0.990 | 0.152 |
| MEmidnightblue | 0.556 | 0.651 | 0.537 | 0.869 | 0.810 | 0.663 | 0.420 | 0.320 | 0.840 | 0.720 |
| MEred | 0.970 | 0.900 | 0.537 | 0.940 | 0.810 | 0.420 | 0.420 | 0.680 | 0.840 | 0.720 |

MDD_CM, major depressive disorder with childhood maltreatment; MDD_nCM, major depressive disorder without childhood maltreatment; FDR-p, False Discovery Rate-adjusted p-value; FC, functional connectivity; HAMA, Hamilton anxiety rating scale; HAMD, Hamilton depression rating scale.
